# Loss of transcriptional regulator of phospholipid biosynthesis alters post-translational modification of Sec61 translocon beta subunit Sbh1 in *Saccharomyces cerevisiae*

**DOI:** 10.17912/micropub.biology.001260

**Published:** 2024-07-12

**Authors:** Jacob M. Miller, Mary E. Tragesser-Tiña, Samantha M. Turk, Eric M. Rubenstein

**Affiliations:** 1 Department of Biology, Ball State University; 2 Diabetes, Obesity, and Complications Therapeutic Area, Eli Lilly and Company; 3 Graduate School of Biomedical Sciences and Department of Developmental Neurobiology, St. Jude Graduate School of Biomedical Science

## Abstract

We recently discovered that disrupting phospholipid biosynthesis by eliminating the Ino2/4 transcriptional regulator impairs endoplasmic reticulum (ER)-associated degradation (ERAD) in *Saccharomyces cerevisiae* , but the mechanism is unclear. Phosphatidylcholine deficiency has been reported to accelerate degradation of Sec61 translocon beta subunit Sbh1 and ERAD cofactor Cue1. Here, we found that, unlike targeted phosphatidylcholine depletion, *INO4* deletion does not destabilize Sbh1 or Cue1. However, we observed altered electrophoretic mobility of Sbh1 in *ino4* Δ yeast, consistent with phospholipid-responsive post-translational modification. A better understanding of the molecular consequences of disrupted lipid homeostasis could lead to enhanced treatments for conditions associated with perturbed lipid biosynthesis.

**Figure 1. Effect of INO4 deletion on electrophoretic migration and degradation of ER proteins:**
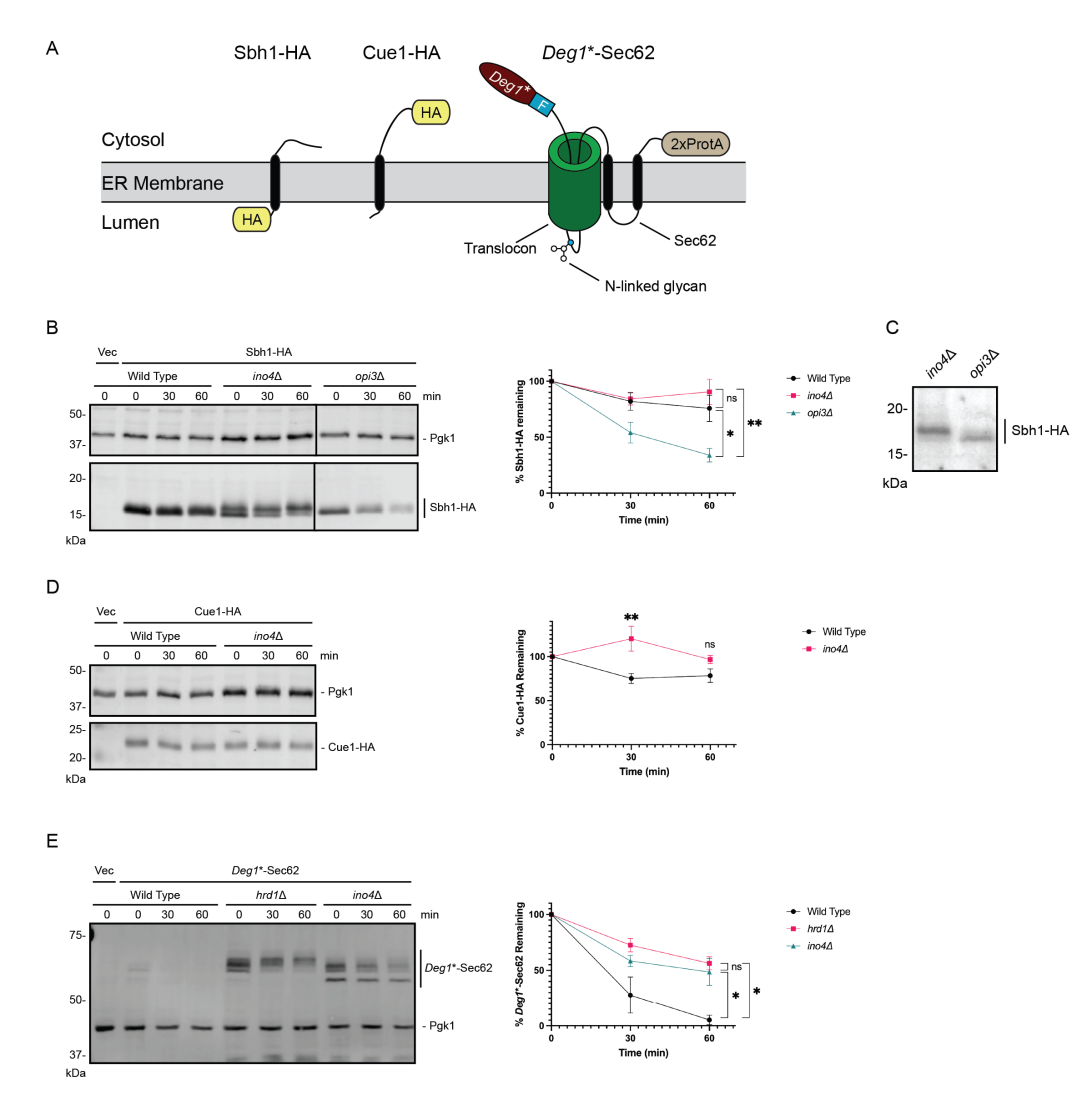
**(A)** ERAD substrates analyzed in this study. Sbh1-HA is a tail-anchored, single-pass transmembrane protein with the HA epitope in the ER lumen (Shyu et al., 2019). Cue1-HA is a single-pass transmembrane protein with the HA epitope in the cytosol (Shyu et al., 2019). *Deg1* *-Sec62 (Runnebohm et al., 2020) is an engineered protein containing, in sequence, *Deg1* * (a modified version of the N-terminal 67 amino acids of transcriptional regulator MATα2), a FLAG epitope (F), the two-transmembrane protein Sec62, and two copies of the *Staphylococcus aureus* Protein A (2xProtA). Upon membrane insertion of the two transmembrane segments of Sec62, a portion of the N-terminal tail loops into and persistently engages the translocon and becomes N-glycosylated. **(B, D, E)** Yeast of the indicated genotypes were transformed with plasmids encoding Sbh1-HA, Cue1-HA, or *Deg1* *-Sec62 (or an empty vector control) and subjected to cycloheximide chase and western blot to detect the ER proteins of interest and the Pgk1 loading control. Means of percent protein remaining of 3-5 biological replicates are plotted. Error bars represent standard error of the mean. ns, not significant. *, p < 0.05, **, p < 0.01. **(C)** Side-by-side comparison of electrophoretic mobility of Sbh1-HA in *ino4* Δ and *opi3* Δ yeast.

## Description

Many diseases are characterized by defects in phospholipid homeostasis (Arendt et al., 2013; Bargui et al., 2021; Kim et al., 2016; Kosicek & Hecimovic, 2013; Li et al., 2006; Mitsuhashi & Nishino, 2011) , and a growing body of literature functionally links phospholipid metabolism and protein degradation (Halbleib et al., 2017; Ho et al., 2020; Hwang et al., 2023; Jonikas et al., 2009; Promlek et al., 2011; Shyu et al., 2019; Thibault et al., 2012; To et al., 2017; Volmer et al., 2013; Xu & Taubert, 2021) . It is therefore important to understand how cellular lipid composition impacts protein dynamics. We recently identified *INO4* in a screen for genes required for endoplasmic reticulum (ER)-associated degradation (ERAD) in *Saccharomyces cerevisiae* (Turk et al., 2023) . *INO4* encodes one subunit of the heterodimeric Ino2/4 master transcriptional regulator of phospholipid biosynthetic genes (Ambroziak & Henry, 1994; Henry et al., 2012) . *INO4* deletion stabilizes a broad panel of ERAD substrates of both major yeast ERAD ubiquitin ligases (Hrd1 and Doa10) without globally impairing the ubiquitin-proteasome system (Turk et al., 2023) . The precise molecular mechanism(s) underlying ERAD disruption by *INO4* deletion remain(s) to be elucidated.

Loss of *INO4* broadly disrupts lipid homeostasis, as Ino2/4 promotes expression of genes required for synthesis of phosphatidylcholine, phosphatidylinositol, and phosphatidylethanolamine, among other phospholipids. Rescue of the *ino4* Δ ERAD defect by supplementation with phospholipid metabolites and intermediates whose uptake or synthesis are controlled by Ino2/4-regulated genes confirmed the degradation impairment is linked to altered phospholipid metabolism (Turk et al., 2023) . Genetic disruption of several lipid biosynthetic pathways (including those specifically responsible for phosphatidylcholine, phosphatidylinositol, and sterol synthesis) impairs ERAD, indicating ER protein quality control is highly sensitive to alterations in membrane composition (Turk et al., 2023) .

We found loss of the phospholipid methyltransferase Opi3 (required for phosphatidylcholine synthesis) significantly stabilizes *Deg1* *-Sec62, a model translocon-clogging ERAD substrate of the Hrd1 ubiquitin ligase (Turk et al., 2023) . Conversely, others have observed *OPI3* deletion *accelerates* Doa10-dependent ERAD of a panel of transmembrane ER proteins, including Sec61 translocon beta subunit Sbh1 and ERAD cofactor Cue1 ( Figure 1A ) (Shyu et al., 2019) . We reasoned that depletion of either Sbh1 or Cue1 might explain the profound ERAD defect in yeast lacking Ino4.

We therefore performed cycloheximide chases and western blots to analyze degradation of Sbh1-HA and Cue1-HA in *ino4* Δ yeast. As previously reported, we observed destabilization of the tail-anchored, single-pass, transmembrane Sbh1-HA in phosphatidylcholine-deficient *opi3* Δ yeast ( Figure 1B ). By contrast, loss of *INO4* did not alter Sbh1-HA degradation kinetics. However, a modified, higher molecular-weight species of Sbh1-HA reproducibly accumulated in *ino4* Δ yeast, and to a more modest extent in *opi3* Δ yeast, relative to wild type yeast ( Figure 1B, 1C). We speculate slowed migration reflects phosphorylation, as multiple proteomic analyses have identified three phosphorylated residues of Sbh1 (Lanz et al., 2021; MacGilvray et al., 2020; Swaney et al., 2013; Zhou et al., 2021) . Additional analyses will be required to definitively characterize the nature of Sbh1 modification. We did not observe destabilization of the single-pass transmembrane Cue1-HA in *ino4* Δ yeast ( Figure 1D ). On the contrary, Cue1-HA was marginally stabilized in *ino4* Δ yeast, consistent with broad ERAD impairment by this mutation. Finally, we reproduced our previous observation that loss of *INO4* stabilizes and impedes post-translational modification of the model translocon-clogging Hrd1 substrate, *Deg1* *-Sec62 ( Figure 1E ).

As *INO4* deletion does not destabilize either HA-tagged Sbh1 or Cue1, our results suggest the ERAD defect in Ino2/4-deficient yeast is not due to depletion of either protein. A precise mechanism by which altered phospholipid biosynthesis in *ino2* Δ or *ino4* Δ yeast impacts ERAD remains elusive. We previously observed that *INO4* deletion does not globally inhibit ER translocation (Turk et al., 2023) . However, phospholipid-sensitive alteration in post-translational modification of a translocon subunit might subtly or selectively perturb translocation in a manner that compromises ERAD. Indeed, recent work indicates phosphorylation alters the conformation of the Sbh1 N-terminus and promotes translocation of a small subset of ER-targeted proteins, modestly impacting the ER proteome (Barbieri et al., 2023; Yadhanapudi et al., 2024) . Loss of Ino2/4 broadly impacts expression of genes regulating lipid homeostasis. It is therefore likely that deletion of *INO2* or *INO4* impacts ER physiology in myriad ways, including altered membrane fluidity, disrupted protein-protein interactions, changes in post-translational modifications, and perturbations in structure, localization, or function of ER-resident proteins.

## Methods

**Cycloheximide chase and yeast cell lysis.** Yeast were cultured in Synthetic Defined (SD) growth medium (2% dextrose, 0.67% yeast nitrogen base, 0.002% adenine, 0.004% uracil, 0.002% arginine, 0.001% histidine, 0.006% isoleucine, 0.006% leucine, 0.004% lysine, 0.001% methionine, 0.006% phenylalanine, 0.005% threonine, and 0.004% tryptophan), lacking uracil or leucine (for plasmid selection). Cycloheximide chase experiments were conducted as described (Buchanan et al., 2016) . In brief, 2.5 OD _600_ units (1 OD _600_ unit is equivalent to 1 mL of yeast at an OD _600_ of 1.0) per time point were harvested by centrifugation and resuspended in fresh medium. Cycloheximide was added to a final concentration of 250 µg/mL. 2.4-OD _600_ aliquots were collected 0, 30, and 60 minutes following cycloheximide addition and transferred to stop solution (10 mM sodium azide, 0.25 mg/mL bovine serum albumin). Proteins were extracted using the alkaline lysis method (Kushnirov, 2000; Watts et al., 2015) .

**Western blotting.** Western blotting was conducted as described (Watts et al., 2015) . Proteins were separated by SDS-PGE and transferred to polyvinylidene difluoride (PVDF) membranes at 20 V for 1 hr at 4°C. Membranes were blocked in Tris-buffered saline (TBS; 50 mM Tris, 150 mM NaCl) with 5% skim milk powder for 1 hr at room temperature or overnight at 4°C. Antibody incubations were conducted for 1 hr at room temperature in TBS supplemented with 1% Tween 20 (TBS/T) and 1% skim milk powder. Each antibody incubation was followed by three five-minute, room-temperature washes with TBS/T. To detect HA-tagged proteins (Sbh1-HA and Cue1-HA), mouse anti-HA.11 primary antibody (Clone 16B12; BioLegend) was used at a dilution of 1:1,000. To detect Pgk1, mouse anti-Pgk1 primary antibody (Clone 22C5D8; LifeTechnologies) was used at a dilution of 1:20,000. Primary antibody incubations were followed by incubation with AlexaFluor-680-conjugated rabbit anti-mouse secondary antibody (LifeTechnologies) at a dilution of 1:20,000. The C-terminal tandem *Staphylococcus aureus* Protein A tags on *Deg1* *-Sec62 were directly detected by AlexaFluor-680-conjugated rabbit anti-mouse secondary antibody (LifeTechnologies) at a dilution of 1:20,000. Membranes were imaged using the Li-Cor Odyssey DLx Imaging System and quantified using ImageStudio software (Li-Cor). To determine percent protein remaining, the ratio of signal intensity for each protein of interest (Sbh1-HA, Cue1-HA, or *Deg1* *-Sec62) to the signal intensity of Pgk1 at each time point was compared to this ratio at 0 minutes for each culture.

**Statistical analysis.** Statistical analyses were performed using GraphPad Prism (version 10). For experiments depicted in Figures 1B and 1E, means of percent protein remaining at 60 minutes were evaluated by one-way ANOVA followed by Holm-Šídák multiple comparison tests. For the experiment depicted in Figure 1D, means of percent remaining at 30 or 60 minutes were evaluated by unpaired, two-tailed *t-* tests.

## Reagents

**Yeast strains used in this study.**

**Table:** 

**Name**	**Genotype**
VJY474	*MATa his3* Δ *1 leu2* Δ *0 met15* Δ *0 ura3* Δ *0 ino4* Δ *::kanMX4*
VJY476 (alias BY4741)	*MATa his3* Δ *1 leu2* Δ *0 met15* Δ *0 ura3* Δ *0*
VJY511	*MATa his3* Δ *1 leu2* Δ *0 met15* Δ *0 ura3* Δ *0 hrd1* Δ *::kanMX4*
VJY1071	*MATa his3* Δ *1 leu2* Δ *0 met15* Δ *0 ura3* Δ *0 opi3* Δ *::kanMX4*

All strains are congenic with BY4741 (Tong et al., 2001) .

**Plasmids used in this study.**

**Table:** 

**Name**	**Alias**	**Description**	**Source or Reference**
pVJ27	pRS316	Empty vector (CEN, *URA3* , *AmpR* )	(Sikorski & Hieter, 1989)
pVJ40	pRS315	Empty vector (CEN, *LEU2* , *AmpR* )	(Sikorski & Hieter, 1989)
pVJ317		*Deg1* *-Sec62 driven by *MET25* promoter in pRS316 backbone (CEN, *URA3* , *AmpR* ). *Deg1* *-Sec62 is *Deg1* * (first 67 amino acids of MATα2 with F18S and I22T mutations), FLAG epitope, Sec62, and two copies of *Staphylococcus aureus* Protein A.	(Rubenstein et al., 2012)
pVJ667	pGT181	Cue1-HA driven by *CUE1* promoter in pRS315 backbone (CEN, *LEU2* , *AmpR* )	(Shyu et al., 2019)
pVJ668	pGT183	Sbh1-HA driven by *SBH1* promoter in pRS315 backbone (CEN, *LEU2* , *AmpR* )	(Shyu et al., 2019)
